# Comparison of Seasonal Soil Microbial Process in Snow-Covered Temperate Ecosystems of Northern China

**DOI:** 10.1371/journal.pone.0092985

**Published:** 2014-03-25

**Authors:** Xinyue Zhang, Wei Wang, Weile Chen, Naili Zhang, Hui Zeng

**Affiliations:** 1 Department of Ecology, College of Urban and Environmental Sciences and the Key Laboratory for Earth Surface Processes of the Ministry of Education, Peking University, Beijing, China; 2 Key Laboratory for Cyclic Economy, School of Urban Planning and Design, Shenzhen Graduate School, Peking University, Shenzhen, China; 3 State Key Laboratory of Vegetation and Environmental Change, Institute of Botany, Chinese Academy of Sciences, Beijing, China; Argonne National Laboratory, United States of America

## Abstract

More than half of the earth's terrestrial surface currently experiences seasonal snow cover and soil frost. Winter compositional and functional investigations in soil microbial community are frequently conducted in alpine tundra and boreal forest ecosystems. However, little information on winter microbial biogeochemistry is known from seasonally snow-covered temperate ecosystems. As decomposer microbes may differ in their ability/strategy to efficiently use soil organic carbon (SOC) within different phases of the year, understanding seasonal microbial process will increase our knowledge of biogeochemical cycling from the aspect of decomposition rates and corresponding nutrient dynamics. In this study, we measured soil microbial biomass, community composition and potential SOC mineralization rates in winter and summer, from six temperate ecosystems in northern China. Our results showed a clear pattern of increased microbial biomass C to nitrogen (N) ratio in most winter soils. Concurrently, a shift in soil microbial community composition occurred with higher fungal to bacterial biomass ratio and gram negative (G-) to gram positive (G+) bacterial biomass ratio in winter than in summer. Furthermore, potential SOC mineralization rate was higher in winter than in summer. Our study demonstrated a distinct transition of microbial community structure and function from winter to summer in temperate snow-covered ecosystems. Microbial N immobilization in winter may not be the major contributor for plant growth in the following spring.

## Introduction

More than half of the earth's terrestrial surface currently experiences seasonal snow cover and soil frost [1], . In these ecosystems, some soil microorganisms are likely protected by the snowpack during winter. With adequate depth and density [3], [4], the heat-insulation snowpack creates a relatively warmer habitat with liquid water films around soil particles [5], [6]. Other soil microorganisms may develop their physiological adaption to survive the chilly environments [7]. Therefore, despite the freezing air temperature, winter catabolic processes of the soil microbial community, detected through biogenic CO_2_ production, can still make a significant contribution to annual ecosystem fluxes across a wide variety of seasonally snow-covered ecosystems [8], [9], [10], [11], [12]. However, the influence of temporal variations in soil anabolic processes (i.e. microbial biomass accumulation) on annual N cycling is not well understood in these ecosystems [13], [14], [15].

Recent studies have reported peak microbial biomass C (MBC) and N (MBN) in late winter, followed by a quick decline when soil temperatures rise to 0°C at alpine and arctic sites [14], [16], [17], [18]. An enlarged N pool was retained in the microbial biomass under the snowpack and subsequently released as a nutrient pulse when soils thawed, which may lead to an increase in N availability for plants at the start of the growing season [13], [14], [16], [19]. The microbial N immobilization observed during winter may play a crucial role in ecosystem function, since it could prevent dissolved N produced by fall litter degradation being lost from the ecosystem during a time when plants are mostly inactive [20]. Therefore, in these snow-covered ecosystems, N retained in the soil by microorganisms during winter may be vital for plant nutrient uptake in the following growing season [18], [21], [22], [23], [24], [25].

Soil microbial community can adapt to changing environmental conditions on very short time scales [7], thus changes between summer and winter may be a key control on annual patterns of nutrient cycling and plant N uptake [26]. For instance, in the alpine tundra of Colorado, winter maximal microbial biomass corresponded with increased biomass in the soil fungal community [27], [28]. Fungi was the primary decomposer of plant debris in the territorial ecosystems, as it could release a great number of extracellular enzymes that can digest a wide variety of substrates, even complex organic compound as lignin [29], [30]. Besides, fungi can grow towards nutrient sources and force their hyphae into solid substrates [31], which help fungi to make use of any nutrient source presented in soils. Furthermore, fungi differ from bacteria in N concentrations and storage capabilities [26], [32]. Based on above viewpoints, fungi dominance in winter may have profound effects on soil biogeochemical cycling in the subsequent growing season. In addition, different gram-staining groups of bacteria, categorized by their cell wall composition, were also found to have various substrate preferences and survival strategies [33]. Therefore, the relative dominance of G- to G+ bacteria may differ between winter and summer. However, it remains poorly understood about the links between the seasonal variation of microbial biomass and the relative abundances of fungi and bacteria in snow-covered temperate areas.

As various decomposer microbes differ in their ability/strategy to efficiently use soil organic matter [34], [35], [36], [37], shifts within the community composition may affect decomposition rates. SOC decomposition is the primary pathway where plant fixed CO_2_ is released back into the atmosphere [38], [39], [40]. Along with frequently observed maximal microbial biomass in the winter of seasonally snow-covered ecosystems, the potential SOC mineralization rate (SOCMR, indicating intrinsic substrate use efficiency) may increase, releasing more CO_2_ into the atmosphere when temperatures rise. However, winter potential SOCMR are seldom conducted [41], [42].

Recent studies of winter soil microbial biogeochemical processes have mainly focused on alpine and tundra ecosystems. However, the wintertime conditions in snow-covered temperate areas could differ from those in alpine and tundra areas. In comparison with alpine and tundra, temperate ecosystems experience a shallower snowpack and shorter duration of snow cover [43], [44], which may modify the extent of the N pool and the seasonal dynamics of its soil microbes. Moreover, temperate soil microbial communities are sensitive to climate changes, because the soil remains close to freezing throughout the winter [45], and small changes in winter temperatures may result in large changes in the amount and timing of snow cover [2]. More importantly, many temperate ecosystems are exposed to larger atmospheric N deposition as a result of increased emissions from industrial and agricultural activities [46]. Consequently, this may induce substantial changes in microbial N dynamics and ecosystem nutrient cycling. However, so far little investigation has been conducted on the winter biogeochemical process of soil microbes in temperate ecosystems [47]. Our study aims to explore the seasonal variation of microbial biomass, community composition and SOC mineralization in temperate ecosystems. The following three questions were addressed: (1) what is the C and N retention capacity of the winter soil microbial community in seasonally snow-covered temperate ecosystems? (2) Does microbial community composition shift from summer to winter? (3) What is the microbial function (organic matter decomposition), as indicated by potential SOCMR, in winter? To answer these questions, summer and winter soil samples were collected from the top 10 cm mineral soil in six seasonally snow-covered temperate ecosystems in northern China. The MBC and MBN, microbial community composition and mineralization rate of SOC were determined. We predicted (1) increased MBN immobilization because of relatively low quality substrates in winter (decreased root exudates and increased autumn litter input) may lead to microbial immobilization of N for growth [23], [48]; (2) bacterial PLFAs to dominate in summer and fungal PLFAs in winter because fungi were commonly reported with a higher C to N ratio (targeting recalcitrant substrates in winter) compared with bacteria (targeting labile substrates in summer); and (3) a higher potential SOCMR in winter than summer because fungi were reported to produce more β-1,4-glucosidase (BG) enzyme (involved in C metabolism) than bacteria [49].

## Materials and Methods

### Ethics Statement

The administration of the Saihanba Forestry Center gave permission for this research at each study site. We confirm that the field studies did not involve endangered or protected species.

### Site information

Our study sites were situated at the Saihanba Forestry Center in Hebei Province, North China (117°12′–117°30′ E, 42°10′–42°50′ N, 1400 m a.s.l.), which is a typical forest-steppe ecotone of a temperate area. The climate in this area is semi-arid and semi-humid, with a long and cold freezing period (November-March) and relatively short growing season. Annual mean air temperature and precipitation between 1964 and 2004 were −1.4°C and 450 mm, respectively. The soils in the region are dominated by aeolian sandy soil, together with meadow and swamp soil. Soil has low nutrient content with SOC content ranging from 0.71 to 2.65% and soil total nitrogen (STN) ranging from 0.08 to 0.26%, respectively (Table 1). Soil bulk density (SBD) ranged from 0.65 to 1.06 g cm^−3^; Soil pH varied from 5.8 to 6.5 and 6.0 to 6.6 in the summer and winter, respectively (Table 1). Soil moisture content was calculated on the gravimetric bias. Specifically, 10 g fresh soil samples were dried to a constant weight by an oven at a temperature of 105°C. All the soil and microbial properties were determined by the dry soil weight.

**Table 1:** Site information and soil properties of the ten sampling sites.

Sites	Location	Domain species	SOC (%)	STN (%)	SBD (g cm^−3^)	ST (°C)		pH	
						JUL.	JAN.	JUL.	JAN.
P1	42°24.707′N 117°14.071′E	*Pinus sylvestris*	0.71 ± 0.05	0.08 ± 0.01	0.98 ± 0.00	19.0	−7.3	6.45 ± 0.10	6.31 ± 0.08
P2	42°24.760′N 117°14.771′E	*Pinus sylvestris*	1.26 ± 0.09	0.12 ± 0.01	0.83 ± 0.05	14.7	−12.8	6.34 ± 0.05	6.36 ± 0.04
P3	42°25.079′N 117°15.974′E	*Pinus sylvestris*	1.10 ± 0.12	0.09 ± 0.01	0.85 ± 0.03	14.9	−8.1	6.31 ± 0.06	6.30 ± 0.07
L1	42°24.332′N 117°12.933′E	*Larix principis-rupprechtii*	0.94 ± 0.04	0.09 ± 0.00	1.06 ± 0.01	15.8	−9.4	6.30 ± 0.02	6.36 ± 0.01
L2	42°24.118′N 117°12.722′E	*Larix principis-rupprechtii*	0.95 ± 0.07	0.09 ± 0.01	0.88 ± 0.01	14.5	−9.7	5.94 ± 0.09	6.02 ± 0.09
L3	42°23.911′N 117°19.052′E	*Larix principis-rupprechtii*	1.88 ± 0.17	0.18 ± 0.02	0.74 ± 0.05	13.7	−7.2	5.78 ± 0.06	6.16 ± 0.04
BH	42°23.848′N 117°19.031′E	*Betula platyphylla*	2.65 ± 0.25	0.26 ± 0.04	0.65 ± 0.00	13.7	−7.8	5.92 ± 0.10	6.16 ± 0.04
MA	42°24.729′N 117°14.132′E	*Malus baccata*	2.10 ± 0.15	0.20 ± 0.01	0.72 ± 0.02	16.1	−7.3	6.39 ± 0.07	6.63 ± 0.06
RO	42°24.107′N 117°13.866′E	*Rosa bella*	1.22 ± 0.16	0.13 ± 0.02	0.73 ± 0.07	19.4	−11.2	6.20 ± 0.07	6.46 ± 0.06
CG	42°24.717′N 117°14.107′E	*Leymus chinensis*	1.00 ± 0.04	0.09 ± 0.00	0.88 ± 0.05	19.9	−11.7	6.28 ± 0.05	6.29± 0.09

Values are presented as mean±standard errors. SOC  =  Soil Organic Carbon; STN  =  Soil totoal nitrogen; SBD  =  Soil Bulk Density; ST  =  Soil Temperature; JUL.  =  July; JAN.  =  January.

**Figure 1:**
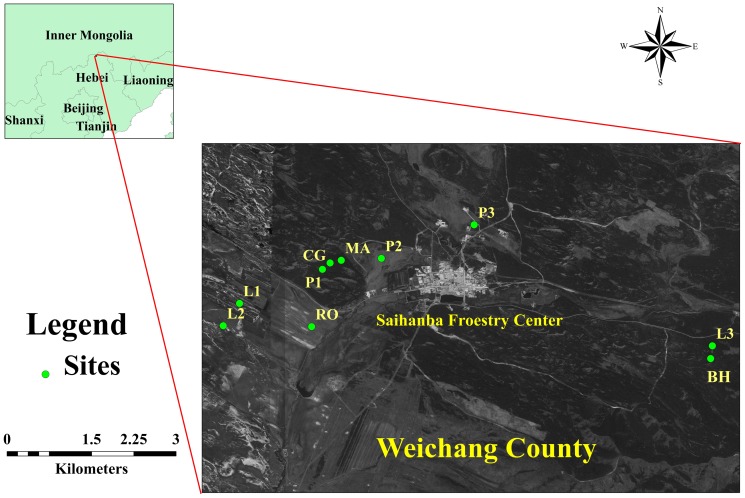
The distribution of sampling sites. P1  =  ∼15-year Mongolia pine; P2  =  ∼25-year Mongolia pine; P3  =  ∼35-year Mongolia pine; L1  =  ∼15-year Prince Rupprecht's larch; L2  =  ∼25-year larch; L3  =  ∼35-year larch; BH  =  Birch; MA  =  Siberian crabapple; RO  =  Solitary rose; CG  =  Meadow grassland.

Primary forests were harvested via large scale industrial logging in the late 1900s and have been replaced by secondary forests and plantations. This site contains the largest area of plantation forests in China, the dominant species are *Pinus sylvestris* var. *mongolica* (Mongolia pine) and *Larix principis-rupprechtii* (Prince Rupprecht's larch). The Mongolia pine and larch herbaceous layers are similar, composed by *Radix Sanguisorbae*, *Thalictrum aquilegifolium L*., *Agrimonia pilosa Ledeb*, and *Carex stenophylla Wahleub*. The secondary forest mainly consists of *Betula platyphylla* (birch) with an herbaceous layer of *Agrimonia pilosa Ledeb*and *Radix Sanguisorbae*. In addition, shrublands dominated by *Rosa bella Rehd. et Wils* (solitary rose) and *Malus baccata* (Siberian crabapple) and meadow grasslands are also very common. The solitary rose herbaceous layer consists of *Leymus chinensis*. The Siberian crabapple herbaceous layer is dominated by *Veronica linariifolia*, *Galium verum*, *Heteropappus hispidus*, *Trollius chinensis*, and *Bupleurum chinense*. The meadow grassland is zonal vegetation dominated by *L. chinensis*. Due to the sparse understory species of the forest sites and simple species composition of the grasslands and shrublands, we did not consider the potential impacts of vegetation diversity on microbial processes. The distribution of sampling sites was shown in Fig. 1, which was drawn by ArcMap (ArcGIS 10.0, Esri Inc., California, USA) with ancillary site information.

### Study design and methods

Because of the significant effect of stand age on ecosystem C and N dynamics [50], we took forest age into consideration in the two coniferous plantation sites, namely Mongolia pine and Prince Rupprecht's larch. Three replicates were selected for each of three age classes (shown in Table 1) in Mongolia pine (sites P1, P2, and P3) and larch (sites L1, L2, and L3). We also selected three replicates for the birch stand (site BH), each of the two shrublands Siberian crabapple (site MA) and solitary rose (site RO) and one meadow grassland (site CG); 30 plot samples in total (Table 1). The area of each plot was 20 × 20 m. All sampling sites were less than 10 km away from each other (Fig. 1) to ensure similar climatic conditions.

All experiments were performed on upper 10 cm mineral soils at five random locations from each plot in July 2010 and January 2011. The two months we chose had the highest (July) and lowest (January) air temperature according to the local climate records of recent decade, representing two most stable seasons of the years, namely summer and winter. This approach has been commonly applied to investigate seasonal dynamics [18], [28], [51]. Under this circumstance, we assumed that the two single months can represent their respective seasons, and that changes over these two seasons in the soil microbial community may be of greater magnitude than differences within the same season or from year to year [52]. In summer, soil was taken by 5.8-cm diameter soil cores, passed through a 2-mm sieve to remove plant litter and roots and homogenized. In winter, after the thickness of snow cover measured by a steel ruler, snows were swept away with a shovel before sampling. The snow thickness varied from 4-16 cm in different sampling sites (data not shown). After that, frozen soils were collected using axe or drill and the upper materials were cut by knife. The processed soils were immediately transported to the laboratory (less than 30 min away) for subsequent analysis.

*In situ* continuous measurements of soil temperature were conducted at 30-min intervals with StowAway loggers (Onset Comp. Corp., Bourne, MA, USA) inserted into the soil at a depth of 5 cm. Extractable ammonium and nitrate nitrogen (NH_4_^+^ and NO_3_^−^) were measured simultaneously in 2 mol/L KCl solution (1∶5 w/v) [53] using a Lachat Flow Injection Analyzer (Lachat Instruments, Milwaukee, WI). The amount of extractable ammonium and nitrate nitrogen were taken as total inorganic N. SOC was measured by the potassium dichromate oxidation method with ground air-dried soils [54] while STN was measured by Kjeldahl method [55]. Because of the minor variation of SOC and STN in our study sites over a year (data not shown), we only measured the values in summer time (July) and did not take the difference between summer and winter into consideration. Soil pH was determined using a pH meter (Denver, US) on a 1∶1 (w/v) air-dried soil to distilled de-ionized water slurry.

### MBN and MBN

MBC and MBN were measured by the chloroform fumigation extraction (CEF) method [56]. Two replicate samples, one un-fumigated and one fumigated with alcohol-free CHCl_3_ for 24 h and then extracted with 0.5 mol/L K_2_SO_4_ (1∶2.5 w/v). MBC and MBN were calculated as the difference of C and N between fumigated and un-fumigated soil extraction, which was estimated using the dichromate oxidation and titration method and Kjeldahl digestion, respectively. To avoid variations in CFE values due to the choice of conversion factor (corrects for the incomplete release and extraction of microbial biomass following CEF method), no conversion factor was used for fumigation efficiency as conducted by Edwards and Jefferies (2013) [18], since it could vary across different seasons as well as among different sampling sites [57]. Soil dissolved organic N (DON) was measured from the initial un-fumigated extraction [18].

### Phospholipid fatty acid (PLFA) analysis

PLFA analysis was used to determine the total microbial biomass and assess microbial community composition. In brief, PLFAs were extracted by a mixture of chloroform-methanol-phosphate buffer (1∶2∶0.8) [58]. Polar lipids in the initial soil extracts were separated from neutral and glycolipids by elution with 5 ml chloroform and 10 ml acetone followed by 5 ml methanol. The polar lipid fraction was used to perform mild alkaline methanolysis. The peak area of each resulting fatty acid methyl ester was recorded on the chromatogram for each sample before identification. Peaks were identified by chromatographic retention time and a standard qualitative mix that ranged from C9 to C30 using a microbial identification system (Microbial ID Inc., Newark, DE). The fatty acid 18∶2ω6, 9 was recognized as the fungal biomarker [59]. Bacterial biomass was quantified as the sum of i14∶0, i15∶0, a15∶0, 16∶1ω9, 16∶1ω7, i17∶0, a17∶0, cy17∶0, 17∶0, and cy19∶0 [60]. G+ bacteria were marked by i15∶0, a15∶0, i16∶0, a16∶0, i17∶0, and a17∶0 [59]. The mono-unsaturated and cyclopropyl saturated peaks 16∶1ω5, 16∶1ω9, 17∶1ω9, cy17∶0, 18∶1ω11, and cy19∶0 were used as indicators for G- bacteria [59], [61], [62]. We also calculated the total lipids as an indicator of microbial biomass [63].

### Potential SOCMR

We used a laboratory incubation experiment to measure the SOCMR through detection of CO_2_ emission [41], [64]. Approximately 25 g of fresh soil were placed into 250 ml glass gas tight jars and incubated at 25°C for 3 d. Respired CO_2_ was captured by a connecting vial with 5 ml 1 mol/L NaOH and determined by titration with 1 mol/L HCl. Potential SOCMR were expressed as the amount of CO_2_-C released per hour per gram soil dry weight. Because the winter soil temperature was below 0°C, it was unable to measure *in situ* mineralization activity using the laboratory incubation method. Thus, we conducted the potential SOCMR in winter using the same temperature as the summer (25°C, being the suitable condition for soil microbial growth in the field). Considering the condition for winter samples was quite different from the *in situ* environment, we indirectly estimated winter mineralization of SOC using the *Q_10_* coefficient (increase in reaction rate per 10°C increase in temperature) obtained from our previous research in the same study sites [12].
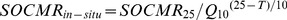
(1)


Where *SOCMR_in-situ_* was the mineralization rate of SOC at an *in situ* temperature measured in winter, *SOCMR*_25_ was the mineralization rate of SOC at the room temperature of 25°C, and *T* was the winter soil temperature at 5 cm depth.

### Statistical analysis

We used the averaged value of the subsamples in each plot to conduct statistical analysis. Statistical analyses were performed using SPSS (ver. 18.0, SPSS Inc., Chicago, IL, USA). Repeated measures analysis of variance (ANOVA) was used to determine the effects of seasons and dominate vegetation types on soil N concentrations, microbial biomass, microbial community composition and potential SOCMR. Besides, ANOVA was also used to determine the effect of stand age on above soil and microbial properties. Furthermore, simple correlation analysis was conducted to explore the relationships between microbial community related parameters and soil physical and chemical properties. In all cases, differences of *P* < 0.05 were regarded as statistically significant. Results were displayed as mean ± standard error.

Besides, we analyzed the PLFAs data by principal components analysis (PCA) to determine whether the PLFA signatures of microbial community varied between summer and winter. PCA was performed on 21 different PLFAs indentified from all the samples with concentration larger than 0.005 fraction (ratio of moles individual lipid to moles total lipid biomass) using R 3.0.1 (R Core Team, 2013). Statistical differences among PLFAs data were assessed using multi-response permutation procedures (MRPP) [65], [66]. MRPP is a nonparametric procedure for testing the hypothesis of no differences between two or more pre-existing groups [67]. *P* value evaluated the significant differences due to chance, where A value described within-group homogeneity compared to random expectation [67]. An A value equal to 1 was found when all items within a group are identical; when heterogeneity within groups equaled expectation by chance A  =  0.

In addition, inverse Simpson index was calculated to describe the microbial diversity obtained from PLFAs data in different seasons as well as in different sampling sites. We preferred this index to other measures of alpha-diversity because it is an indication of the richness in a community with uniform evenness that would have the same level of diversity.

## Results

### Soil physical and chemical properties

**Figure 2:**
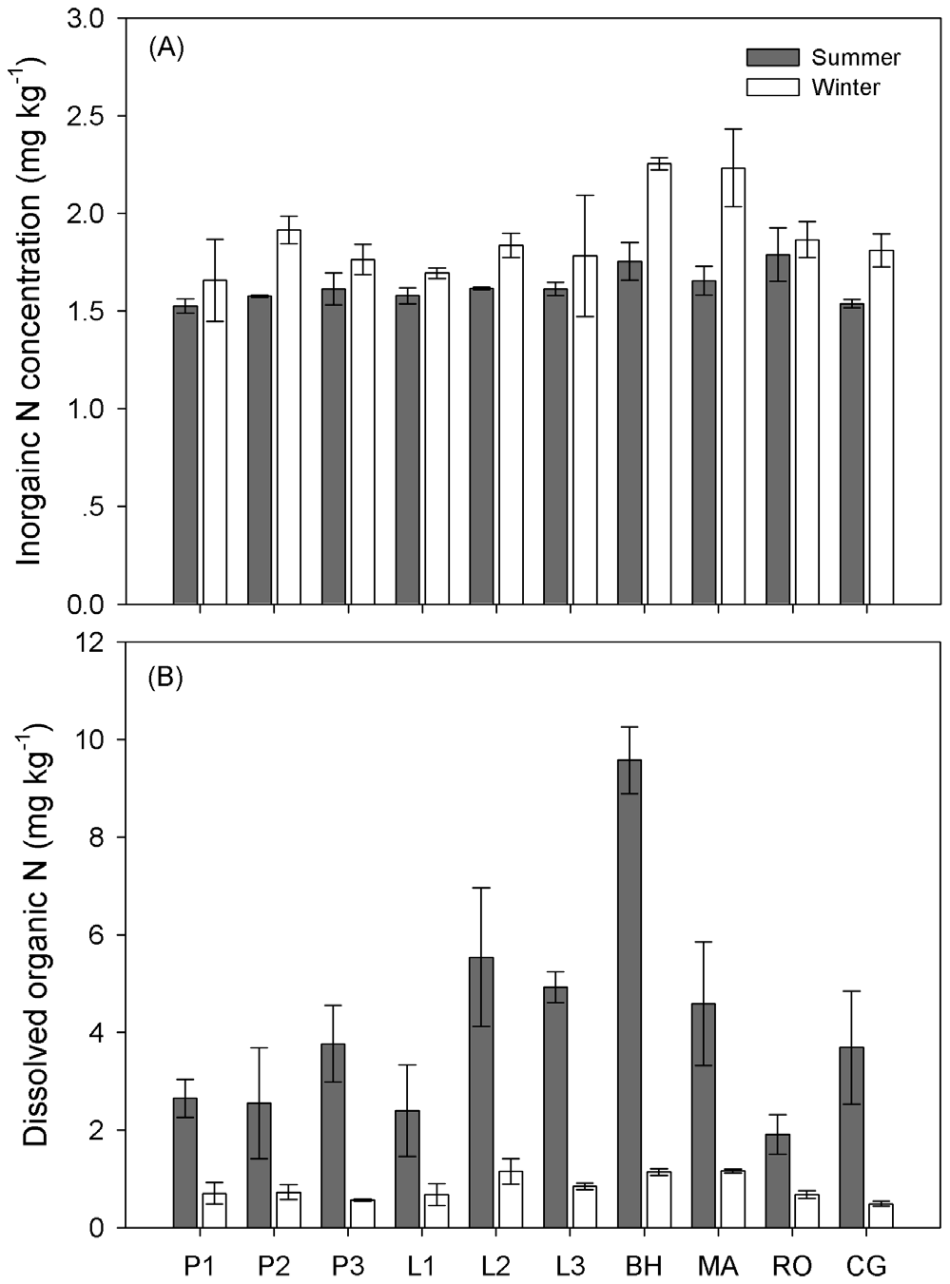
Inorganic nitrogen (N) (A) and dissolved organic N (B) across different sites in summer and winter. Results are presented as mean ± stander error. Support information is presented in Table S1.

In July, soil temperature at 5-cm depth averaged 16.5°C, and decreased to −9.5°C in January across six ecosystems (Table 1). Soil NH_4_^+^-N and NO_3_^−^-N concentrations averaged 1.59 ± 0.02 mg kg^−1^ and 0.03 ± 0.01 mg kg^−1^ in July and 1.86 ± 0.05 mg kg^−1^ and 0.02 ± 0.01 mg kg^−1^ in January, respectively (Table 2). Although total soil inorganic nitrogen (NO_3_^−^-N + NH_4_^+^-N) across all ecosystems was significantly higher in winter than in summer at a statistical level (*P* < 0.001), there was no large difference between them (1.88 ± 0.07 mg kg^−1^ in winter vs. 1.63 ± 0.03 mg kg^−1^ in summer) (Table 3; Fig. 2A; Table S1). DON was significantly higher in summer than in winter, with average value decreasing from 4.76 ± 0.52 mg kg^−1^ in summer to 0.84 ± 0.06 mg kg^−1^ in winter, respectively (Table 3; Fig. 2B; Table S1). Although DON significantly differed across different ecosystem types, no significant interaction between seasons and sampling sites was observed (Table 3; Fig. 2B).

**Table 2:** Soil inorganic nitrogen (NH_4_^+^-N and NO_3_^−^-N) concentrations, microbial biomass carbon to nitrogen (MBC/N) ratio and fungi to bacteria biomass (F/B) ratio of the ten sampling sites.

Sites	NH_4_^+^-N (mg kg^−1^)		NO_3_^−^ -N (mg kg^−1^)		MBC/N		F/B	
	JUL.	JAN.	JUL.	JAN.	JUL.	JAN.	JUL.	JAN.
P1	1.48 ± 0.03	1.66 ± 0.21	0.04 ± 0.01	NT	15.90 ± 4.57	49.26 ± 5.18	0.024 ± 0.001	0.126 ± 0.011
P2	1.54 ± 0.00	1.92 ± 0.07	0.04 ± 0.00	NT	23.54 ± 5.07	41.58 ± 6.25	0.023 ± 0.002	0.136 ± 0.039
P3	1.60 ± 0.08	1.76 ± 0.08	0.02 ± 0.01	NT	30.68 ± 6.82	55.04 ± 5.31	0.033 ± 0.002	0.132 ± 0.011
L1	1.57 ± 0.04	1.69 ± 0.03	0.01 ± 0.00	NT	8.89 ± 2.49	31.48 ± 1.57	0.054 ± 0.013	0.115 ± 0.018
L2	1.61 ± 0.01	1.84 ± 0.06	0.01 ± 0.00	NT	35.36 ± 3.22	68.38 ± 16.33	0.083 ± 0.004	0.075 ± 0.003
L3	1.60 ± 0.03	1.78 ± 0.31	0.00 ± 0.00	NT	33.87 ± 8.95	47.83 ± 7.87	0.079 ± 0.003	0.097 ± 0.019
BH	1.73 ± 0.96	2.25 ± 0.03	0.02 ± 0.00	NT	15.34 ± 2.46	31.97 ± 4.28	0.056 ± 0.007	0.096 ± 0.006
MA	1.63 ± 0.08	2.23 ± 0.20	0.03 ± 0.01	NT	41.79 ± 13.95	33.74 ± 1.18	0.060 ± 0.004	0.076 ± 0.007
RO	1.73 ± 0.13	1.85 ± 0.10	0.06 ± 0.01	NT	9.70 ± 1.09	31.59 ± 7.83	0.073 ± 0.006	0.078 ± 0.004
CG	1.45 ± 0.03	1.67± 0.08	0.08 ± 0.01	NT	9.25 ± 3.72	33.69 ± 1.06	0.070 ± 0.008	0.066 ± 0.004

Values are presented as mean±standard errors. NT =  not detectable.

**Table 3:** The repeated measure ANOVA results of soil and microbial properties tested in this study.

ANOVA results	IN	DON	MBC	MBN	MB	F/B	G−/G+	Potential SOCMR
Season	*P* < 0.001	*P* < 0.001	*P* < 0.001	*P* = 0.015	*P* = 0.509	*P* < 0.001	*P* < 0.001	*P* < 0.001
Sites	*P* = 0.044	*P* = 0.032	*P* < 0.001	*P* = 0.002	*P* < 0.001	*P* = 0.509	*P* < 0.001	*P* < 0.001
Interaction	*P* = 0.297	*P* = 0.086	*P* = 0.048	*P* = 0.121	*P* = 0.390	*P* < 0.001	*P* < 0.001	*P* = 0.017

IN  =  inorganic nitrogen; DON  =  dissolved organic nitrogen; MB  =  microbial biomass; G−/G+  =  gram negative to positive bacteria biomass ratio; SOCMR  =  soil organic carbon mineralization rate.

### Soil MBC and MBN

**Figure 3:**
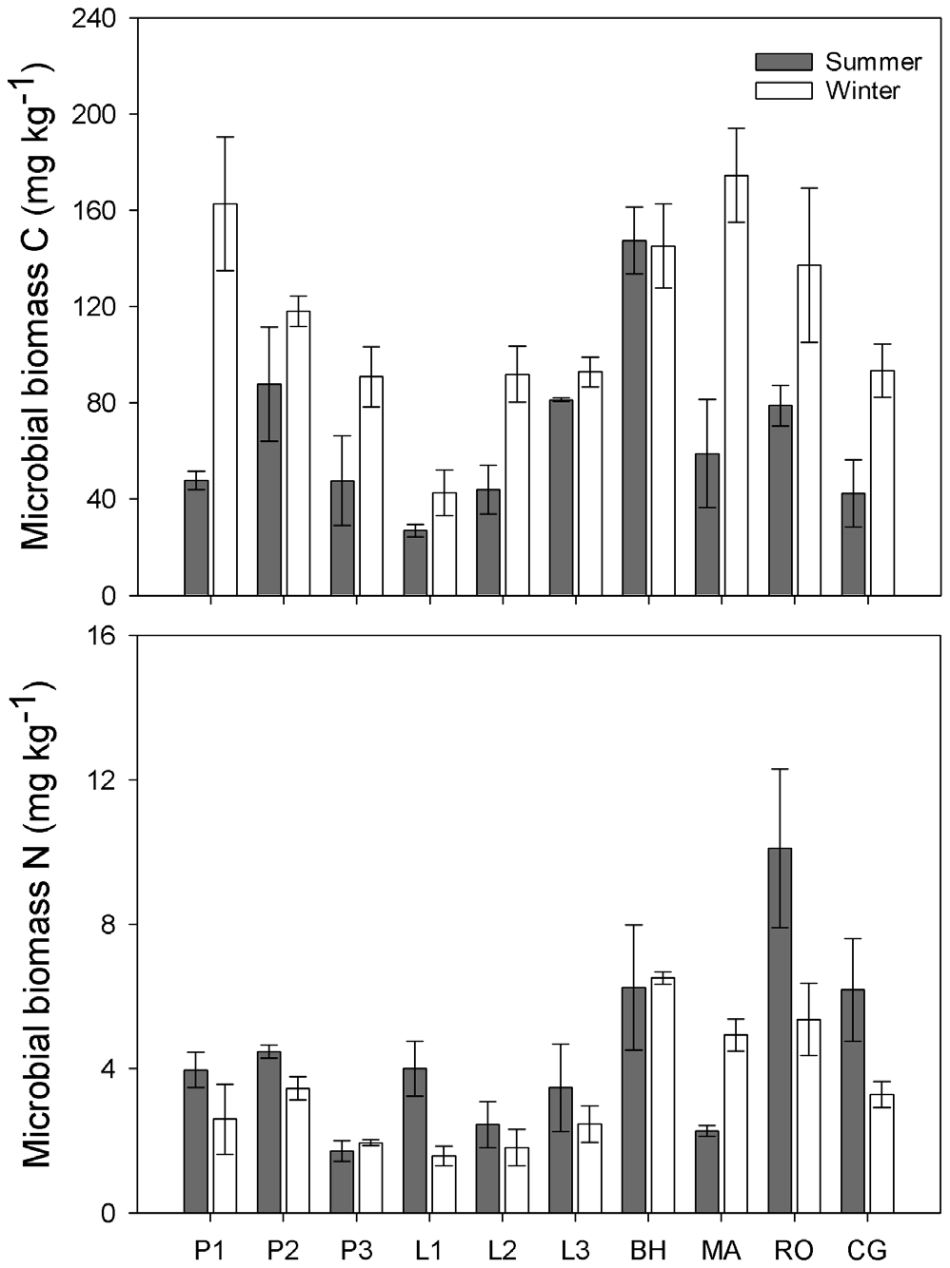
Microbial biomass carbon (C) (A) and nitrogen (N) (B) across different sites in summer and winter. Results are presented as mean ± stander error. Support information is presented in Table S1.

Across all the sampling sites, soil microbes showed a significantly higher MBC (*P* < 0.001) while lower MBN (*P*  =  0.015) in winter than in summer (Table 3; Fig. 3). MBC consistently increased from 66.1 ± 7.4 mg kg^−1^ in summer to 112.1 ± 8.5 mg kg^−1^ in winter on average, except for site BH. MBN in most of the sites were lower in winter than in summer (4.5 ± 0.5 mg kg^−1^ in summer vs. 3.3 ± 0.3 mg kg^−1^ in winter) (Table S1). Both MBC and MBN varied significantly across different sites as well as in different seasons (Table 3). Besides, the interaction between seasons and sampling sites also showed significant effect on MBC but not MBN (Table 3). MBC significantly correlated with MBN in both summer (R^2^  =  0.27, *P*  =  0.020) and winter (R^2^  =  0.52, *P* < 0.001). MBC to MBN (MBC/N) ratio varied from 9.25 ± 3.72 to 41.79 ± 13.95 in summer, and 31.48 ± 1.57 to 68.38 ± 16.33 in winter (Table 2). The MBC/N ratio consistently increased from summer to winter across all the sampling sites, except for site MA, where a slight decrease was found (Table 2). Stand age exhibited no significant influence on MBC/N ratio for both summer and winter.

### Microbial community composition

**Figure 4:**
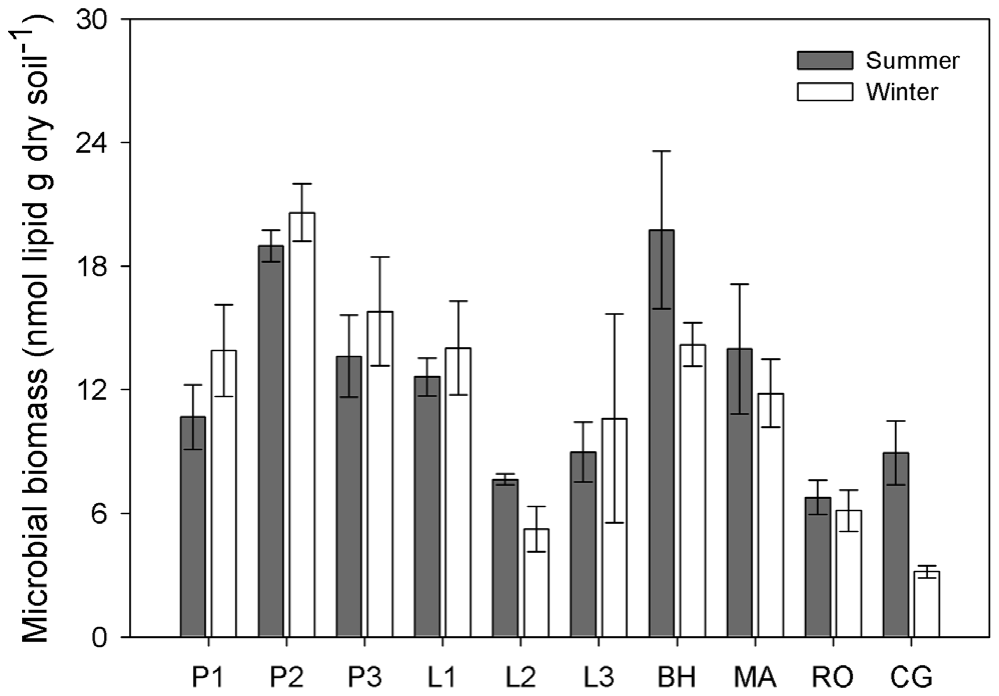
Total microbial biomass indicated by PLFAs across different sites in summer and winter. Results are presented as mean ± stander error. Support information is presented in Table S1.

**Figure 5:**
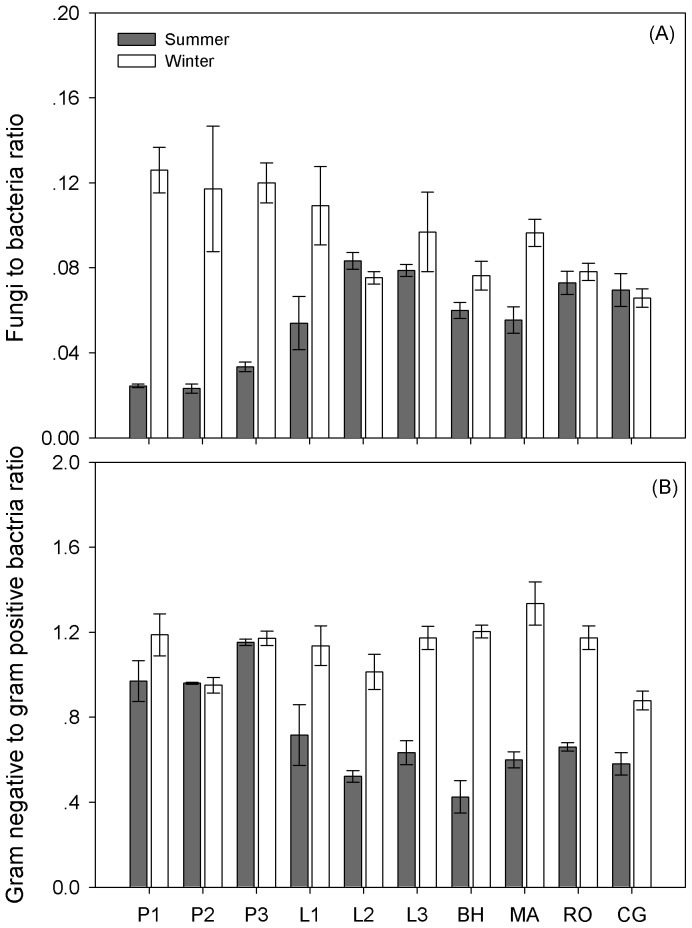
Soil fungal to bacterial PLFAs ratio (A) and gram negative to gram positive bacterial PLFAs ratio (B) across different sites in summer and winter. Results are presented as mean ± standard error. Support information is presented in Table S1.

Microbial biomass indicated by total PLFAs showed no significant difference between summer and winter (*P*  =  0.509), but varied significantly among different sampling sites (*P* < 0.001) (Table 3; Fig. 4; Table S1). In addition, microbial community composition shifted with higher fungal biomass abundance in winter (Table 3; Fig. 5A). The bacterial community biomass was not significantly different between summer and winter (*P*  =  0.802) across all the sites, as well as the G+ bacterial community biomass (*P*  =  0.050). However, G- bacterial biomass significantly increased in winter (*P* < 0.001; varying from 1.98 ± 0.35 nmol PLFAs g dry soil^−1^ in summer to 3.66 ± 0.51 nmol PLFAs g dry soil^−1^ in winter). Thus the G- to G+ bacterial biomass (G−/G+) ratio also exhibited a significant increase in winter (Fig. 5B; Table S1). In summer, fungal to bacterial biomass (F/B) ratio was negatively correlated with soil pH value (R^2^  =  0.43, *P*  =  0.036), whereas, in winter, F/B ratio was positively correlated with total microbial biomass indicated by PLFAs (R^2^  =  0.78, *P*  =  0.001) (Table 4). G−/G+ ratio was negatively correlated with soil temperature only in winter (R^2^  =  0.62, *P*  =  0.007) (Table 4).

**Table 4:** The correlation between soil properties (listed in the row) and microbial community parameters (listed in the line).

Correlation Matrix		MBC/N		MB		F/B		G−/G+	
		JUL.	JAN.	JUL.	JAN.	JUL.	JAN.	JUL.	JAN.
ST	R	−0.508	0.183	−0.508	0.134	−0.015	0.075	0.076	0.790**
	*P*	0.134	0.612	0.134	0.712	0.967	0.836	0.835	0.007
pH	R	0.138	−0.255	0.138	0.189	−0.665**	0.340	0.595	0.033
	*P*	0.704	0.477	0.704	0.601	0.036	0.337	0.070	0.928
SOC	R	0.515	−0.374	0.515	0.164	0.222	−0.234	−0.520	0.482
	*P*	0.128	0.287	0.128	0.651	0.537	0.516	0.123	0.158
STN	R	0.485	−0.408	0.485	0.145	0.226	−0.258	−0.551	0.507
	*P*	0.155	0.242	0.155	0.688	0.530	0.490	0.099	0.135
DON	R	0.406	0.170	0.406	−0.018	0.280	−0.340	−0.581	0.421
	*P*	0.244	0.639	0.244	0.961	0.433	0.336	0.078	0.225
IN	R	0.099	−0.380	0.099	0.094	0.363	−0.354	−0.448	0.361
	*P*	0.785	0.279	0.785	0.797	0.303	0.316	0.194	0.305

R  =  correlation coefficient; ** and * represents *P* < 0.01 and *P* < 0.05 respectively.

**Figure 6:**
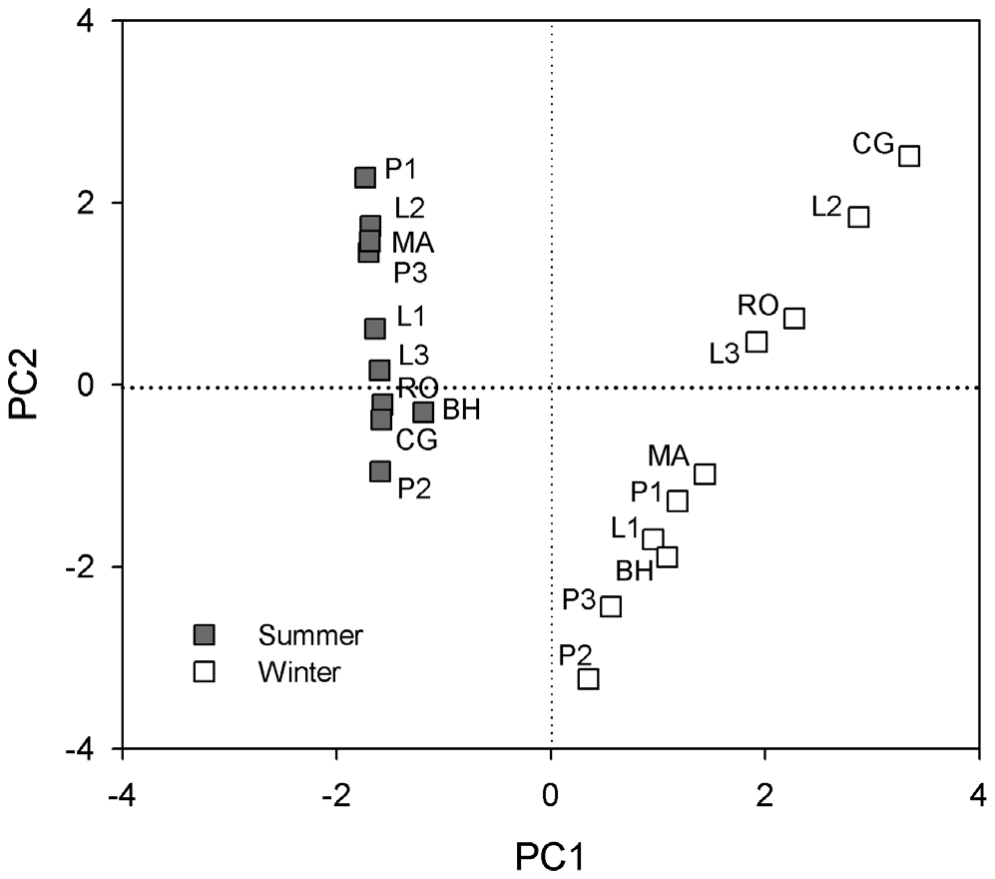
Principal component analysis (PCA) of PLFA signatures (mol percentages) from soil samples collected in summer and winter. PC1 and PC2 explained 96% and 2% variance of the PLFAs data, respectively.

Principle component analysis (PCA) also showed the differentiation in microbial community structure between seasons (Fig. 6). The first principle component axis (PC1) alone could explain as much as 96% of the variance in the PLFAs data. There was no evidence of difference in PLFAs data among different sampling sites (MRPP: *P*  =  0.693, A  =  0), however, significant difference of PLFAs was observed between summer and winter (MRPP: *P*  =  0.001, A  =  0.56). Microbial diversity was significantly lower in winter (4.76) than that in summer (8.59) indicated by inverse Simpson index (*P* < 0.001), and differed in each sampling site (*P* < 0.001). In all sampling sites, microbial diversity showed a decrease trend from summer to winter (Table S2). Stand age exerted no significant effect on microbial community composition or diversity.

### Potential SOCMR

**Figure 7:**
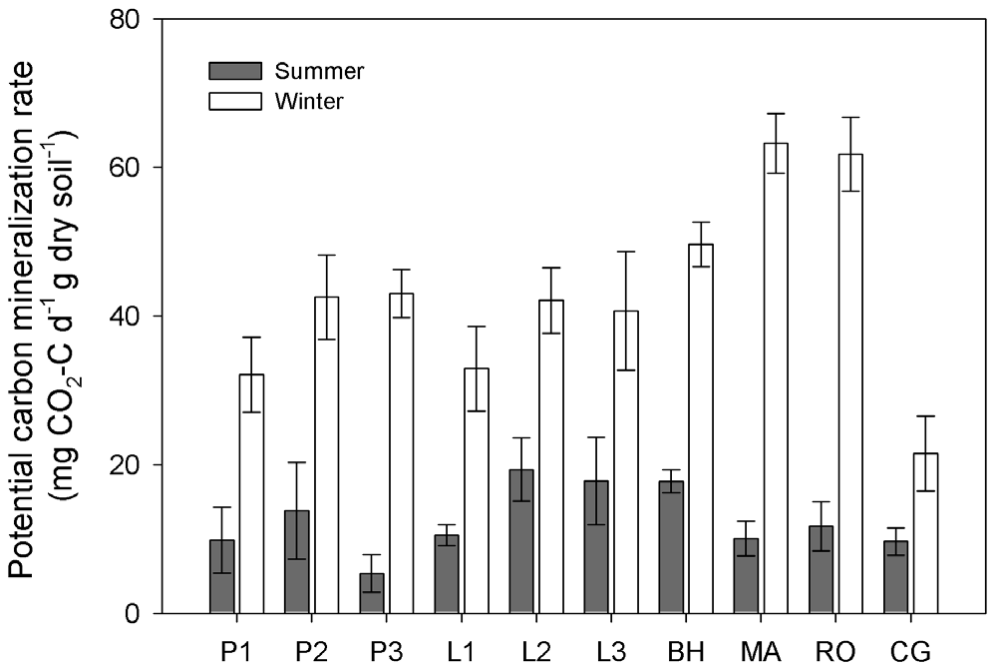
Potential carbon mineralization rates across different sites in summer and winter. Results are presented as mean ± standard error. Support information is presented in Table S1.

The C emission rate ranged from 5.31 ± 2.50 mg CO_2_-C d^−1^ g dry soil^−1^ in summer to 63.22 ± 4.02 mg CO_2_–C d^−1^ g dry soil^−1^ in winter under the same incubation temperature, showing a significantly higher potential SOCMR for winter microbes than summer ones (Table 3; Fig. 7; Table S1). However, when applied the Q_10_ value to model the *in situ* winter SOCMR, the average *in situ* mineralization rate was 0.26 mg CO_2_–C d^−1^ g dry soil^−1^, quite lower than the averaged summer ones (12.57 mg CO_2_–C d^−1^ g dry soil^−1^). No significant effect of stand age was observed on potential SOCMR for both summer and winter.

## Discussion

Multiple studies in alpine and arctic tundra ecosystems have reported active microbial metabolism under snow-cover during winter [26], [27], [28], [50], [68], [69]. However, until now little has been known about winter microbial biogeochemical processes in temperate areas. This study determined the soil microbial biomass, community composition and mineralization rate of SOC in a variety of seasonally snow-covered temperate ecosystems.

It was commonly thought that relatively low quality substrates may lead to microbial immobilization of N for growth [23], [48]. Therefore, we expected increased MBN immobilization because of decreased root exudates and increased autumn litter input in winter when plants were inactive. However, in our study area, there existed a clear pattern of increased MBC to MBN ratio in winter across almost all of the ecosystems, mainly through increased MBC (Fig. 3A) and reduced MBN (Fig. 3B). Non-increased microbial N pool in winter was unexpected and contrasted with the findings from alpine and tundra areas [26] and corresponding to a temperate beech forest soil [20]. Exception was only found in site MA, where MBN obviously increased from summer to winter (Fig. 3B). However, there was no soil or microbial properties measured in this study seemed to explain this exception. The lower levels of microbial N immobilization in our temperate winter soils indicate a smaller or no nitrogen pulse from winter soil microbes in the following spring. The discrepancy may be due to the differences of snow cover depth and duration. Across our studied sites, the snowpack was as thin as 4–16 cm and the duration of snow cover was short. Snowpack less than 30 cm depth could not effectively decouple soil temperatures from the atmosphere [70], [71], [72], and thus resulting in lower microbial N immobilization.

As total microbial PLFAs changed negligibly (Fig. 4), the microbial biomass was stable across different seasons. However, the increased fungal dominance seemed to partly explain the increased C and decreased N uptake. The positive relationship between fungal abundance and the MBC to MBN ratio has been observed in a previous global analysis [73]. Fungi were commonly reported with a higher C to N ratio (targeting recalcitrant substrates) compared with bacteria (targeting labile substrates) [74], [75]. In summer, warmer temperature can increase root exudates (labile substrates) than in winter [76] and fresh plant litter input had a relatively lower C to N ratio. Therefore, we expected bacterial PLFAs to dominate. In contrast, in winter, a lot of leaf litter with a relative high C to N ratio accumulated on the soil floor and likely became a major substrate source for fungi. We did observe increased fungal PLFAs (*P* < 0.001), but no significant variation in bacterial PLFAs was found between summer and winter (*P*  =  0.809). Because it is common for soil microbes to preferentially use simple organic compounds over complex polymers [77], [78], winter substrate usage may be largely confined to microbial recycling of dead microbial cells and hyphae [79] or endogenous metabolism (the breakdown of living cell constituents/storage compounds for maintenance). Therefore, the dominance of fungi in winter may be largely due to their resistance to freeze-thawing events [80], and not to substrate preference. Fungal to bacterial PLFA ratios increased in winter across all sites (Fig. 5A). This result was consistent with those obtained in alpine and tundra ecosystems [27], . As seasonal shifts in microbial species composition may also occur at fine taxonomic scales [81], further research into the finer microbial community composition is warranted.

Although no significant variation occurred in the common bacterial PLFAs, we observed a significant increase of G- bacteria in cold environments (*P* < 0.001); in contrast, G+ bacterial PLFAs did not change under winter conditions (*P*  =  0.050), which was consistent with previous observation in late winter tussock tundra soil [82]. G- bacteria can more easily access the soil aqueous phase [33], thus the structure of the cell wall may help it to survive and grow in lower temperatures. Although G- bacteria seemed to take advantage over G+ at colder temperature, correlation analysis across different sampling sites showed that G- to G+ bacterial biomass ratio positively related to winter soil temperature (Table 4). Researches also suggested that G+ and G- bacteria differ in their patterns of substrate preference [83] with the former being dominant in soils with low substrate availability [60] and the latter in those with high availability of easily decomposable substrate [84]. Because the winter microbial substrate source was unknown, whether or not the increase in G- bacteria corresponds with the change of substrate preference across sites remains unclear.

A shift in microbial community composition along with fungal dominance in winter may subsequently change the enzyme production by the preference of different microbes and may ultimately influence the potential SOC mineralization rate. Fungi have been reported to produce more BG enzyme (involved in C metabolism) than bacteria [51]. As the BG enzyme was considered as an overall indicator of microbial activity [83], [85], we expected a higher potential SOCMR in winter than summer. The result was consistent with our expectation, indicating without the temperature constraints substrate use efficiency was higher for winter microbes than the summer ones. However, *in situ* SOCMR was much lower in winter than in the summer. Although low winter temperature constrained the actual substrate mineralization rate, once temperature rise, the mineralization rate may experience a sharp increase, along with high C metabolism enzyme pool suggested by fungi dominance, and eventually leading to more CO_2_ released back to atmosphere. The rapid increase of soil respiration from late winter to early spring has been observed in our previous field study conducted in the same ecosystems [12].

## Conclusion

In summary, the trend of increased microbial C and decreased N uptake in winter dominate across all six seasonally snow-covered temperate ecosystems. Therefore, the N pool retained in the microbial biomass under the snowpack may not be major source for spring plant nutrient demand. The higher MBC to MBN ratio in winter was partly connected to the shift in microbial composition to fungal dominance in winter. Because there were significant differences in substrate use, nutrient limitation, and N storage capacity between fungi and bacteria, the changes in winter fungal to bacterial biomass ratios might substantially alter annual patterns of nitrogen cycling in seasonally snow-covered temperate ecosystems. Although *in situ* C mineralization was low due to the temperature constraints in winter, greater potential SOCMR indicated a higher intrinsic substrate use efficiency of winter microbes than summer ones. Our results suggested significant differences in microbial community structure and function between summer and winter. Considering the future changes in winter climate and N deposition in temperate areas, more detailed investigations of seasonal dynamics in soil microbial biomass, community structure and function including spring and autumn are urgently needed in the scenario of global changes.

## Supporting Information

Table S1 **Detailed summer (JUL.) and winter (JAN.) values of soil and microbial properties in** **Figure 2** **; 3; 4; 5B; 7.** P1  =  ∼15-year Mongolia pine; P2  =  ∼25-year Mongolia pine; P3  =  ∼35-year Mongolia pine; L1  =  ∼15-year Prince Rupprecht's larch; L2  =  ∼25-year larch; L3  =  ∼35-year larch; BH  =  Birch; MA  =  Siberian crabapple; RO  =  Solitary rose; CG  =  Meadow grassland. DON  =  dissolved organic nitrogen, MBC  =  microbial biomass carbon, MBN  =  microbial biomass nitrogen, MB  =  microbial biomass.
(DOCX)

Table S2 **The inverse Simpson index of summer (JUL.) and winter (JAN.) PLFAs data in each sampling site.**
(DOCX)
