# Supplementary material for: Comparison of Seasonal Soil Microbial Process in Snow-Covered Temperate Ecosystems of Northern China
Source: PLoS One. 2014 Mar 25;9(3):e92985. doi: 10.1371/journal.pone.0092985 (PMC3965484; doi:10.1371/journal.pone.0092985)
Supplement: Table S1 — Detailed summer (JUL.) and winter (JAN.) values of soil and microbial properties in Figure 2 ; 3; 4; 5B; 7. P1 = ∼15-year Mongolia pine; P2 = ∼25-year Mongolia pine; P3 = ∼35-year Mongolia pine; L1 = ∼15-year Prince Rupprecht's larch; L2 = ∼25-year larch; L3 = ∼35-year larch; BH = Birch; MA = Siberian crabapple; RO = Solitary rose; CG = Meadow grassland. DON = dissolved organic nitrogen, MBC = microbial biomass carbon, MBN = microbial biomass nitrogen, MB = microbial biomass. (DOCX) [file pone.0092985.s001.docx]

| Sites | Inorganic N (mg kg^-1^) | | DON (mg kg^-1^) | | MBC (mg kg^-1^) | | MBN (mg kg^-1^) | | MB (nmol lipid g dry soil^-1^) | |
| --- | --- | --- | --- | --- | --- | --- | --- | --- | --- | --- |
|  | JUL. | JAN. | JUL. | JAN. | JUL. | JAN. | JUL. | JAN. | JUL. | JAN. |
| P1 | 1.53 ± 0.04 | 1.66 ± 0.21 | 2.66 ± 0.39 | 0.71 ± 0.22 | 47.7 ± 3.9 | 162.7 ± 27.8 | 4.0 ± 0.5 | 2.6 ± 1.0 | 10.7 ± 1.6 | 13.9 ± 2.2 |
| P2 | 1.58 ± 0.00 | 1.92 ± 0.07 | 2.55 ± 1.13 | 0.73 ± 0.15 | 87.7 ± 23.7 | 118.0 ± 6.4 | 4.5 ± 0.2 | 3.4 ± 0.3 | 19.0 ± 0.8 | 20.6 ± 1.4 |
| P3 | 1.61 ± 0.08 | 1.76 ± 0.08 | 3.77 ± 0.78 | 0.57 ± 0.02 | 47.6 ± 18.7 | 90.9 ± 12.5 | 1.7 ± 0.3 | 1.9 ± 0.1 | 13.6 ± 2.0 | 15.8 ± 2.6 |
| L1 | 1.58 ± 0.04 | 1.69 ± 0.03 | 2.40 ± 0.94 | 0.68 ± 0.22 | 26.9 ± 2.6 | 42.5 ± 9.4 | 4.0 ± 0.8 | 1.6 ± 0.3 | 12.6 ± 0.9 | 14.0 ± 2.3 |
| L2 | 1.62 ± 0.01 | 1.84 ± 0.06 | 5.54 ± 1.42 | 1.16 ± 0.26 | 43.9 ± 10.1 | 91.9 ± 11.7 | 2.4 ± 0.6 | 1.8 ± 0.5 | 7.7 ± 0.3 | 5.2 ±1.1 |
| L3 | 1.61 ± 0.03 | 1.78 ± 0.31 | 4.92 ± 0.32 | 0.85 ± 0.07 | 81.2 ± 0.79 | 92.8 ± 6.1 | 3.5 ± 1.2 | 2.5 ± 0.5 | 8.9 ± 1.4 | 10.6 ±5.1 |
| BH | 1.76 ± 0.10 | 2.25 ± 0.03 | 9.58 ± 0.68 | 1.14 ± 0.07 | 147.4 ± 13.9 | 145.2 ± 17.6 | 6.2 ± 1.7 | 6.5 ± 0.2 | 19.8 ± 3.8 | 14.2 ±1.1 |
| MA | 1.66 ± 0.07 | 2.23 ± 0.20 | 4.59 ± 1.27 | 1.16 ± 0.04 | 58.9 ± 22.5 | 174.6 ± 19.5 | 2.3 ± 0.2 | 4.9 ± 0.4 | 14.0 ± 3.2 | 11.8 ± 1.6 |
| RO | 1.79 ± 0.14 | 1.87 ± 0.09 | 1.91 ± 0.40 | 0.68 ± 0.08 | 78.8 ± 8.5 | 137.1 ± 32.1 | 10.1 ± 2.2 | 5.4 ± 1.0 | 6.8 ± 0.8 | 6.1 ± 1.0 |
| CG | 1.54 ± 0.02 | 1.81± 0.08 | 3.69 ± 1.16 | 0.49 ± 0.05 | 42.2 ± 14.0 | 93.4 ± 11.0 | 6.2 ± 1.4 | 3.3 ± 0.4 | 8.9 ± 1.5 | 3.2 ± 0.3 |

| Sites | G-/G+ | | Potential carbon mineralization rate (mg CO_2_-C d^-1^ g dry soil^-1^) | |
| --- | --- | --- | --- | --- |
|  | JUL. | JAN. | JUL. | JAN. |
| P1 | 0.97 ± 0.10 | 1.19 ± 0.10 | 9.83 ± 4.42 | 32.11 ± 5.05 |
| P2 | 0.96 ± 0.00 | 0.95 ± 0.04 | 13.78 ± 6.52 | 42.52 ± 5.70 |
| P3 | 1.15 ± 0.02 | 1.17 ± 0.03 | 5.32 ± 2.50 | 43.03 ± 3.23 |
| L1 | 0.72 ± 0.14 | 1.14 ± 0.09 | 10.47 ± 1.42 | 32.90 ± 5.70 |
| L2 | 0.52 ± 0.03 | 1.01 ± 0.08 | 19.33 ± 4.25 | 42.10 ± 4.39 |
| L3 | 0.63 ± 0.06 | 1.17 ± 0.05 | 17.81 ± 5.85 | 40.68 ± 7.99 |
| BH | 0.43 ± 0.08 | 1.20 ± 0.03 | 17.75 ± 1.58 | 49.64 ± 2.98 |
| MA | 0.60 ± 0.04 | 1.34 ± 0.10 | 10.04 ± 2.33 | 63.23 ± 4.02 |
| RO | 0.66 ± 0.02 | 1.17 ± 0.06 | 11.69 ± 3.31 | 61.75 ± 4.96 |
| CG | 0.58 ± 0.05 | 0.88 ± 0.04 | 9.65 ± 1.85 | 21.49 ± 5.04 |
