# Supplementary material for: Comparison of Seasonal Soil Microbial Process in Snow-Covered Temperate Ecosystems of Northern China
Source: PLoS One. 2014 Mar 25;9(3):e92985. doi: 10.1371/journal.pone.0092985 (PMC3965484; doi:10.1371/journal.pone.0092985)
Supplement: Table S2 — The inverse Simpson index of summer (JUL.) and winter (JAN.) PLFAs data in each sampling site. (DOCX) [file pone.0092985.s002.docx]

| Sites | P1 | P2 | P3 | L1 | L2 | L3 | BH | MA | RO | CG |
| --- | --- | --- | --- | --- | --- | --- | --- | --- | --- | --- |
| JUL. | 7.48 ± 0.14 | 9.84 ± 0.37 | 7.77 ± 0.77 | 8.62 ± 0.24 | 7.58 ± 0.22 | 8.93 ± 0.79 | 9.90 ± 0.12 | 8.10 ± 0.12 | 8.27 ± 0.22 | 9.38 ± 0.48 |
| JAN. | 5.17 ± 0.63 | 8.36 ± 0.29 | 7.30 ± 0.81 | 6.05 ± 0.89 | 2.20 ± 0.25 | 4.04 ± 1.36 | 5.49 ± 0.72 | 4.34 ± 0.54 | 2.85 ± 0.33 | 1.82 ± 0.08 |
